# Feasibility of gadoxetate disodium enhanced 3D T1 MR cholangiography (MRC) with a specific inversion recovery prepulse for the assessment of the hepatobiliary system

**DOI:** 10.1371/journal.pone.0203476

**Published:** 2018-09-05

**Authors:** Ute Lina Fahlenkamp, Lisa Christine Adams, Sarah Maria Böker, Günther Engel, Minh Huynh Anh, Moritz Wagner, Bernd Hamm, Marcus Richard Makowski

**Affiliations:** Department of Radiology, Charité - Universitätsmedizin Berlin, Berlin, Germany; The First Affiliated Hospital of Nanjing Medical University, CHINA

## Abstract

**Aim:**

To compare the potential of a gadoxetate disodium enhanced navigator-triggered 3D T1 magnetic-resonance cholangiography (MRC) sequence with a specific inversion recovery prepulse to T2-weighted MRCP for assessment of the hepatobiliary system.

**Materials and methods:**

30 patients (12 male, 18 female) prospectively underwent conventional navigator-triggered 3D turbo spin-echo T2-weighted MRCP and 3D T1 MRC with a specific inversion pulse to minimise signal from the liver 30 minutes after administration of gadoxetate disodium on a 1.5 T MRI system. For qualitative evaluation, biliary duct depiction was assessed segmentally following a 5-point Likert scale. Visualisation of hilar structures as well as image quality was recorded. Additionally, the extrahepatic bile ducts were assessed quantitatively by calculation of signal-to-noise ratios (SNR).

**Results:**

The advantages of T1 3D MRC include reduced affection of image quality by bowel movement and robust depiction of the relative position of the extrahepatic bile ducts in relation to the portal vein and the duodenum compared to T2 MRCP. However, overall T1 3D MRC did not significantly (p > 0.05) improve the biliary duct depiction compared to T2 MRCP in all segments: Common bile duct 4.1 vs. 4.4, right hepatic duct 3.6 vs. 4.2, left hepatic duct 3.5 vs. 4.1. Image quality did not differ significantly (p > 0.05) between both sequences (3.6 vs. 3.5). SNR measurements for the hepatobiliary system did not differ significantly (p > 0.05) between navigator-triggered T1 3D MRC and T2 MRCP.

**Conclusions:**

This preliminary study demonstrates that T1 3D MRC of a specific inversion recovery prepulse has potential to complement T2 MRCP, especially for the evaluation of liver structures close to the hilum in the diagnostic work-up of the biliary system in patients receiving gadoxetate disodium.

## Introduction

Anatomical evaluation of the biliary system is of high importance in patients with biliary diseases as well as with intrahepatic or hilar tumours of different aetiologies in the preoperative setting. Magnetic resonance cholangiopancreaticography (MRCP) has proven its utility in a variety of biliary and pancreatic diseases, including choledocholithiasis, congenital anatomic variants, chronic pancreatitis, post-cholecystectomy disorders, and neoplastic duct obstruction. As a high level of diagnostic confidence can be achieved using MRCP at different scanner systems and a different field strengths, this sequence type has contributed to the reduction of the frequency of invasive procedures, especially of direct endoscopic cholangiopancreaticography [1–5].

T2-weighted MRCP with a high spatial resolution is a clinically established MR sequence for visualisation of the pancreatobiliary anatomy. However, a relatively long echo time (TE) is used to acquire this sequence. Therefore, MRCP is susceptible to potential artefacts and pitfalls, e.g. respiratory motion artefacts, susceptibility artefacts by gastric-duodenal gas and artefacts from surrounding fluid-containing structures, especially the small intestine. Additionally, pulsatility from adjacent vascular structures may cause artefacts interfering with luminal contours of the biliary tract thereby causing pseudo-obstruction [6, 7].

Gadoxetate disodium is excreted by hepatocytes into the biliary system with a peak after 10 to 20 minutes [8]. A substantial amount (up to 50%) of gadoxetate disodium is excreted via the biliary system [9]. As a result, the bile and the hepatobiliary system can directly be visualised on T1-weighted sequences, due to the resulting strong T1 shortening effect of the excreted contrast agent [10, 11].

In this study we used in respiratory navigator-gated T1 inversion recovery sequence specifically optimized to visualise the hepatobiliary system with a high contrast. The purpose of the present study was to test the feasibility of gadoxetate disodium enhanced 3D T1 MR cholangiography (MRC) with a specific inversion recovery prepulse for the assessment of the hepatobiliary system, compared to the standard T2 MRCP approach.

## Materials and methods

### Patients

The study was approved by the institutional review board Institutional Review Board (Charité Universitätsmedizin Berlin Ethikkommission, Ethikausschuss 1 am Campus Charite—Mitte). 30 patients (12 men, 18 women, 26–79 years, mean 53 years ± 15) underwent gadoxetate disodium enhanced MRI of the liver after written informed consent to participate in the study. Patients were referred to MRI for evaluation of metastatic disease due to primary cancer of the breast (n = 7), of the thyroid gland (n = 1), of the gastrointestinal tract (n = 2), melanoma (n = 1), extragonadal tumor (n = 1), neuroendocrine tumor (n = 3), angiosarcoma (n = 1), and due to HCC (n = 3). Non-malignant indications (n = 11) were suspected FNH on ultrasound and hemochromatosis. Exclusion criteria were age younger than 18 years, pregnancy, metallic implants or functional devices not eligible for MR examination, claustrophobia, a history of allergic reaction to Gd-EOB-DTPA, and a glomerular filtration rate below 30 ml/min.

### Imaging protocol

MR imaging was performed on a 1.5 T scanner (Avanto, Siemens Medical Solutions, Erlangen, Germany) equipped with a 32-channel body-phased-array coil. Patients underwent the standard liver MR imaging protocol using the hepatocyte specific contrast agent gadoxetate disodium which includes an axial T1-weighted spin echo sequence, an axial fat-saturated T2-weighted turbo spin echo sequence acquired with a 2D navigator for abdominal imaging (2D Prospective Acquisition Correction, PACE), and an axial T1-weighted dual echo sequence. A conventional T2 weighted MRCP was acquired before axial T1 VIBE (volume-interpolated breath-hold) sequences for dynamic imaging before and 15, 55 seconds and 2, 5, 10 and 20 minutes after contrast agent administration and a coronally orientated T1 VIBE sequence for the hepatobiliary phase at least 20 minutes after contrast agent administration.

In addition, a navigator-triggered 3D MRC was performed in the paracoronal plane. Imaging parameters for T2 MRCP and T1 3D MRC are given in Table 1.

**Table 1:** Imaging parameters of the institutional standard T2 MRCP protocol and T1 3D MRC parameters.

Sequence parameters	T2 MRCP	T1 3D MRC
**TR (ms)**	1600	377.70
**TE (ms)**	622	1.79
**FoV read (mm)**	380	380
**FoV phase (%)**	100	100
**Slice thickness (mm)**	1.5	1.5
**Base resolution**	384	384
**Phase resolution (%)**	100	100

MRCP magnetic resonance cholangiopancreaticography MRC magnetic resonance cholangiography TR repetition time TE echo time FoV field of view

### Image analysis

All imaging sequences including the study sequence were analyzed on standard workstations (Centricity PACS, Radiology RA1000, General Electrics).

#### Qualitative evaluation

Biliary duct depiction quality was assessed following a 5-point Likert scale (5: excellent– 4: good– 3: moderate– 2: poor– 1: not visible at all) separately for the following segments: common bile duct (CBD), common hepatic duct (CHD), cystic duct, right and left hepatic duct, first, second, and third order branches.

Impairment of biliary tract assessment by bowel movement was recorded likewise using a 5-point Likert scale with 5 meaning no impairment at all, 4 indicating discrete impairment, 3 indicating moderate impairment, 2 indicating prominent impairment, and 1 meaning cholangiography not diagnostic due to very intense impairment by bowel movement.

Relative position of the central extrahepatic bile ducts to other hilar structures, namely the portal vein and to the duodenum was rated on a 3-point Likert scale (2 –good; 1 –moderate; 0 –other structures not visible at all).

Finally, all-over image quality was rated on a 5-point Likert scale (5 –excellent; 4 –good; 3 –moderate; 2 –poor; 1 –non diagnostic).

### Quantitative evaluation

For quantitative evaluation of the biliary tract depiction, a circular region-of-interest (ROI) was placed within the common bile duct, the common hepatic duct (CHD) and the cystic duct. The size of the ROI was chosen as large as possible, carefully avoiding extrabiliary space. Signal-to-noise ratio (SNR) was calculated dividing signal intensity of the biliary segment by extracorporal signal intensity.

### Statistical analysis

Mean, range and standard deviation were calculated for presentation of the values of SNR as well as for qualitative parameters. Metric T1 3D MRC and T2 MRCP data sets were compared using a student’s unpaired t-Test, with a two-tailed distribution for samples of unequal variance. Ordinal data were compared using the Wilcoxon signed-rank test. A p-value of less than 0.05 was considered statistically significant.

## Results

### Qualitative evaluation

**Fig 1:**
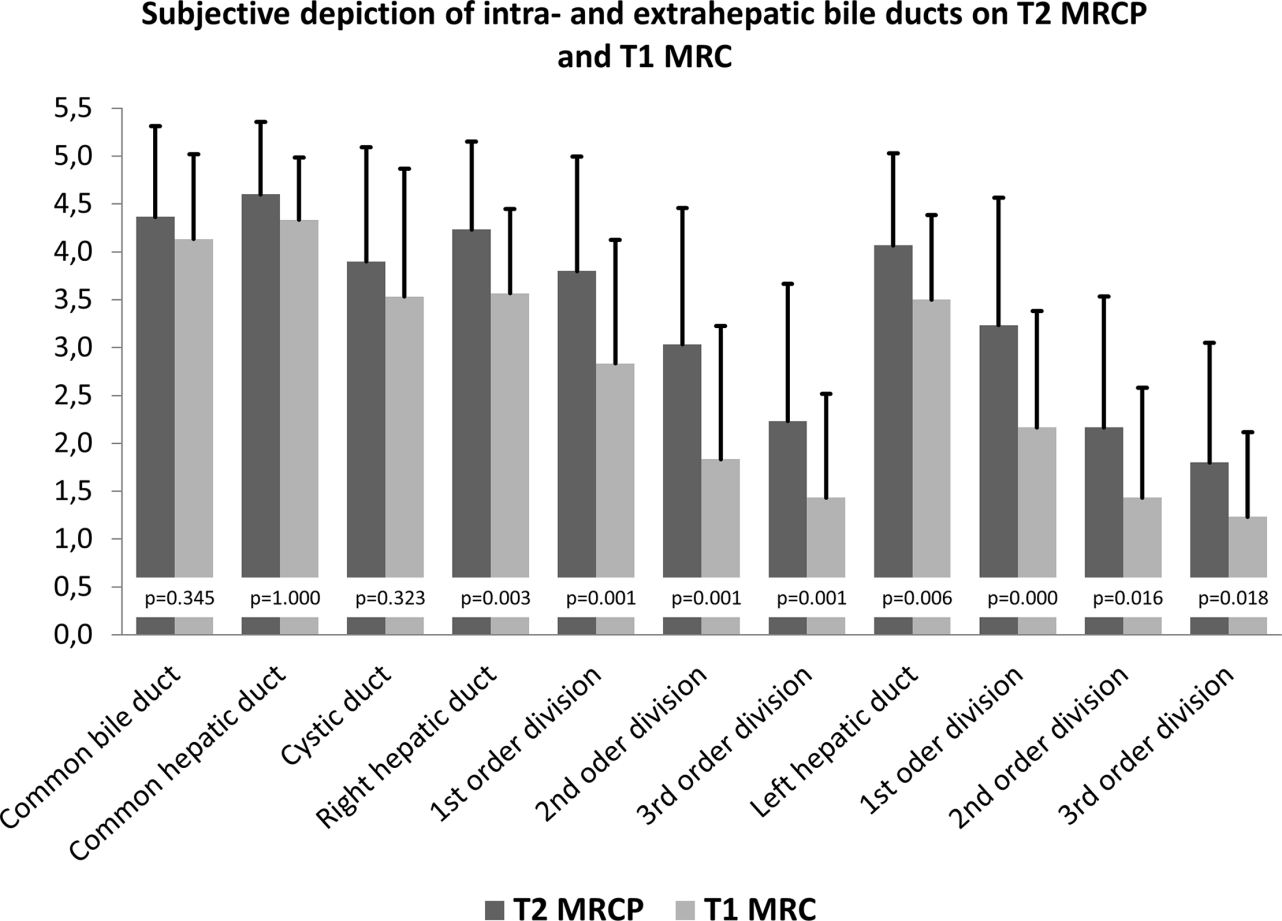
Subjective depiction of intra- and extrahepatic bile ducts on T2 MRCP and T1 3D MRC. For all segments, depiction on T2 MRCP is of higher quality.

Biliary duct depiction showed a high image quality with T2 MRCP than navigator-triggered T1 3D MRC in all segments (see Table 2 and Fig 1).

**Table 2:** Subjective evaluation of biliary duct depiction.

	T1 3D MRC	T2 MRCP	p
Common bile duct	4.1	4.3	0.345
common hepatic duct	4.3	4.5	1.000
cystic duct	3.5	3.8	0.323
Right hepatic duct	3.5	4.1	0.003
Right first order branch	2.7	3.7	0.001
Right second order branch	1.7	2.9	0.001
Right third order branch	1.3	2.1	0.001
Left hepatic duct	3.5	4.0	0.006
Left first order branch	2.1	3.1	0.000
Left second order branch	1.4	2.0	0.016
Left third order branch	1.2	1.7	0.018

Impairment of biliary tract assessment by bowel movement was lower with navigator-triggered T1 3D MRC than with T2 MRCP (4.1 vs. 3.2).

**Fig 2:**
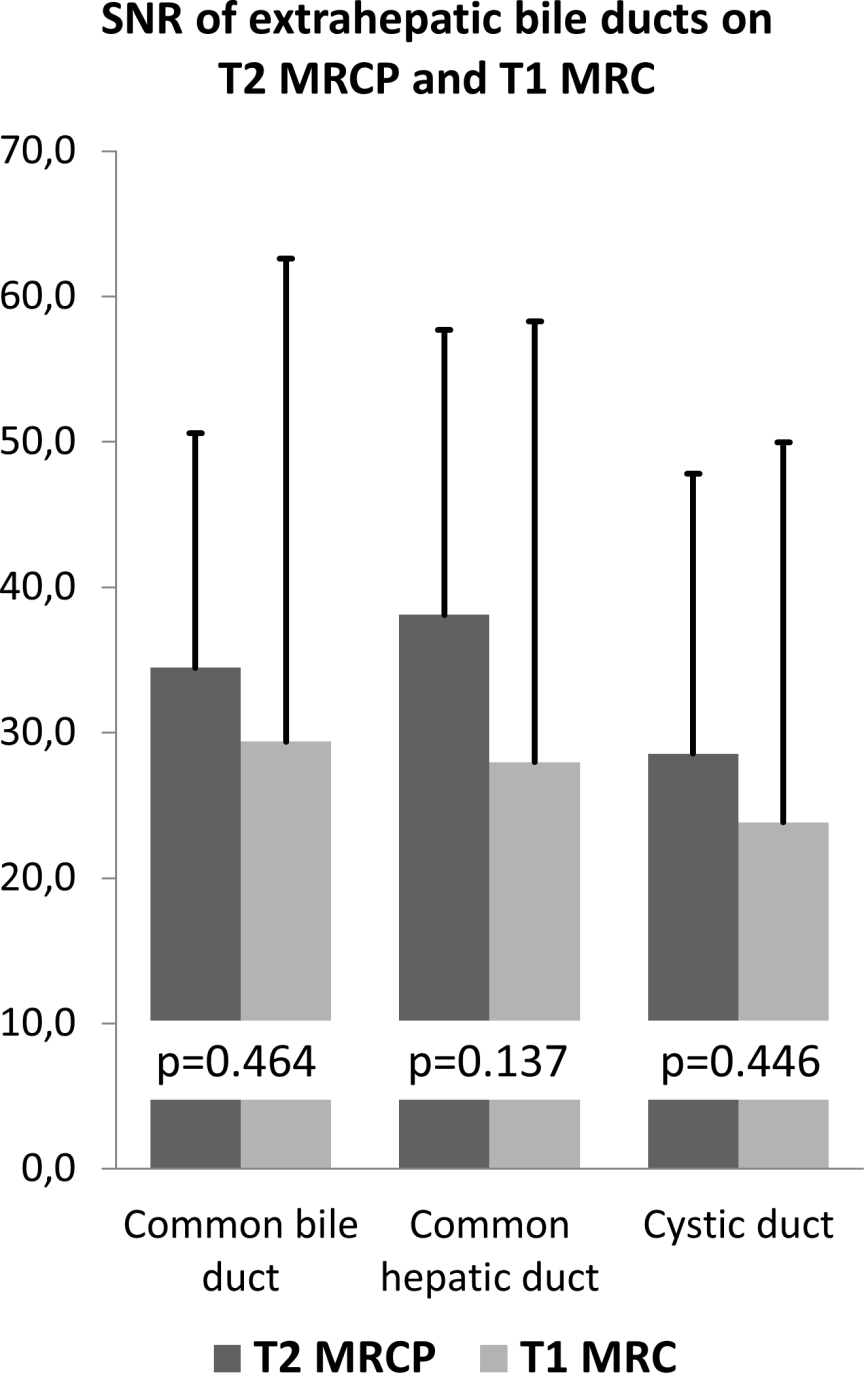
SNR of extrahepatic bile ducts on T2 MRCP and T1 3D MRC. SNR on T2 MRCP is slightly higher in all segments compared to T1 3D MRC.

On navigator-triggered T1 3D MRC, the relative position of the extrahepatic bile ducts compared to the portal vein was robustly depicted (mean 1.8), whereas on T2 MRCP the portal vein was not visualised. Relative position to the duodenum was improved on navigator-triggered T1 3D MRC compared to T2 MRCP even though the effect was not significant (1.7 vs. 1.2, p = 0.114) (see Fig 2).

All-over image quality was comparable for both sequences (3.7 vs. 3.5, p = 0.240).

**Fig 3:**
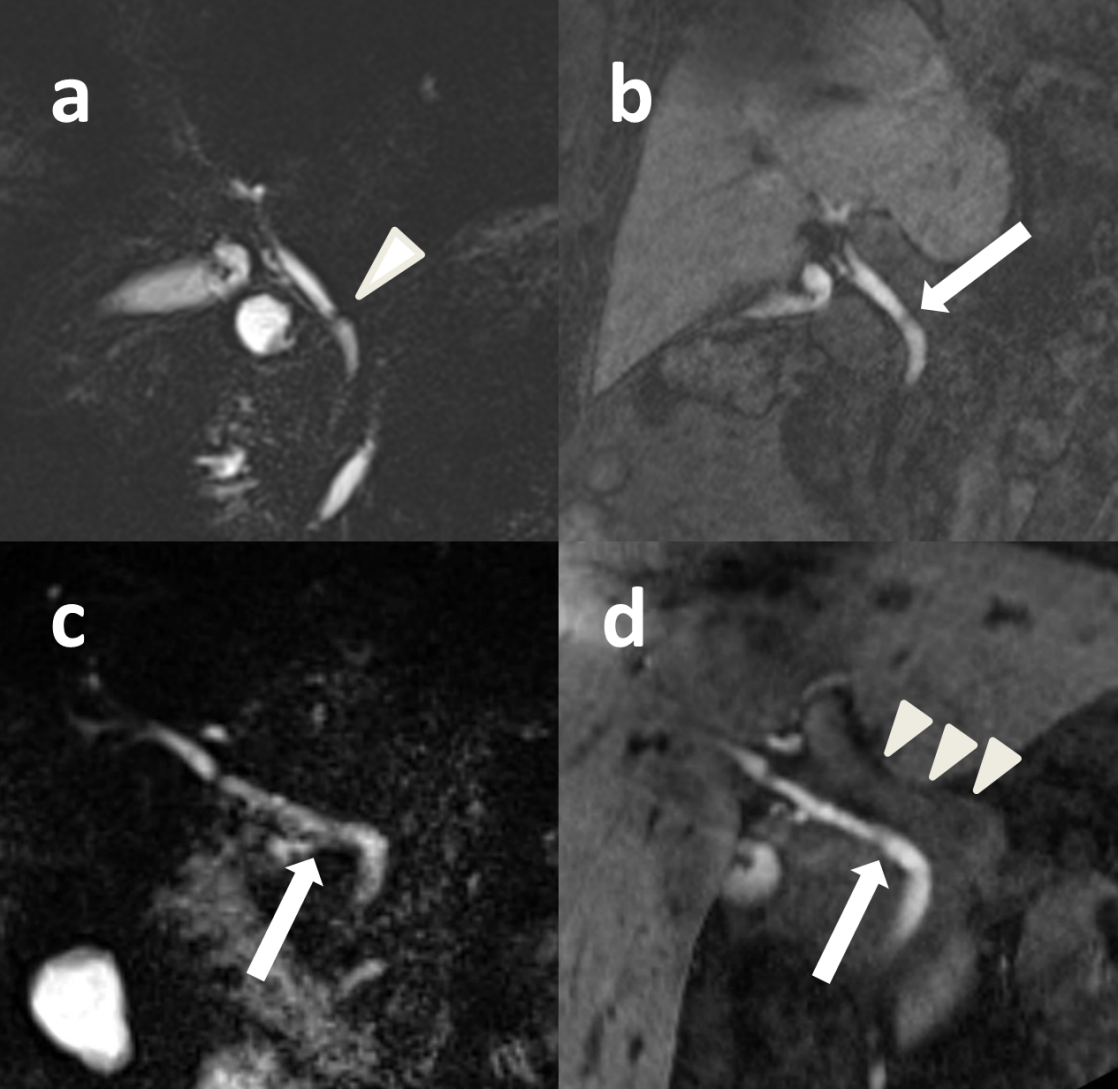
Image examples of T1 3D MRC complementing T2 MRCP. (a,b) In a 75 year-old female patient, on T2 MRCP (a), an artefact simulated a possible stenosis of the common bile duct (white arrowhead). With T1 MRC (b), a relevant stenosis could confidentially be excluded (white arrow). (c, d) In a patient with metastasized breast cancer due to movement of the bowel conspicuity of the central bilary structures was reduced on T2 MRCP Note improved depiction of the proximal part of the common hepatic duct (white arrow) on T1 3D MRC, which is slightly overlayered by duodenal filling on T2 MRCP. Additionally, there is a clear delineation of the portal vein on T1 MRC (white arrowheads).

**Fig 4:**
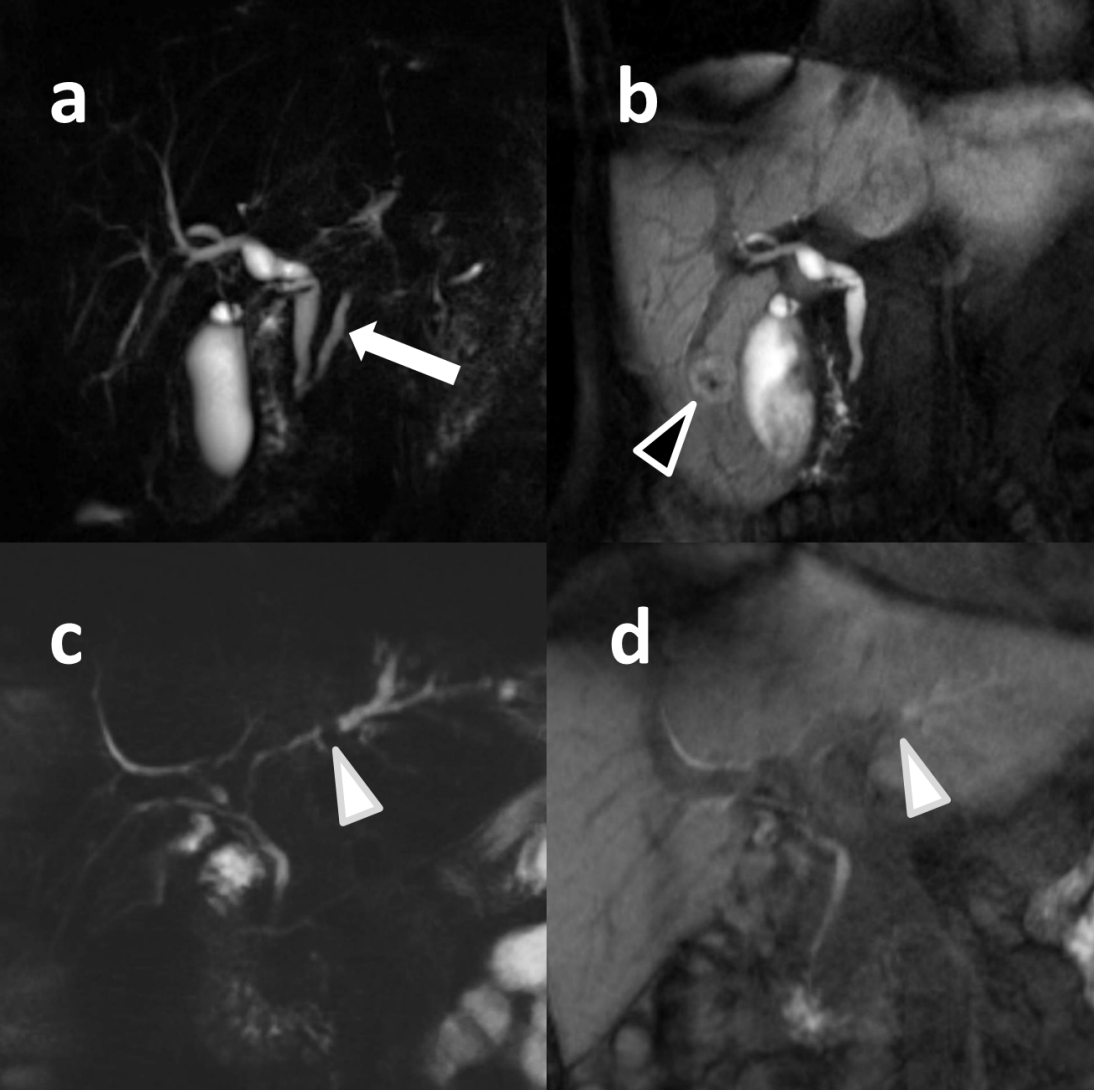
**Maximum intensity projection images of the biliary system (a, c: T2 MRCP, b, d: T1 MRC).** 53 year-old female patient examined due to suspected focal nodular hyperplasia (black arrowhead in T1 MRC, not visible on T2 MRCP). As contrast in T1 MRC is given by gadoxetate disodium, the pancreatic duct visualized on T2 MRCP (white arrow) is not visualized T1 MRC. Please also note improved conspicuity of the intrahepatic bile ducts on T2 MRCP compared to T1 MRC, which can also be seen on in a 30 year-old female patient (c, d) who was examined due to suspected intrahepatic choledocholithiasis (white arrowheads) which could be verified with MR.

Image examples are given on Figs 3 and 4.

### Quantitative evaluation

**Fig 5:**
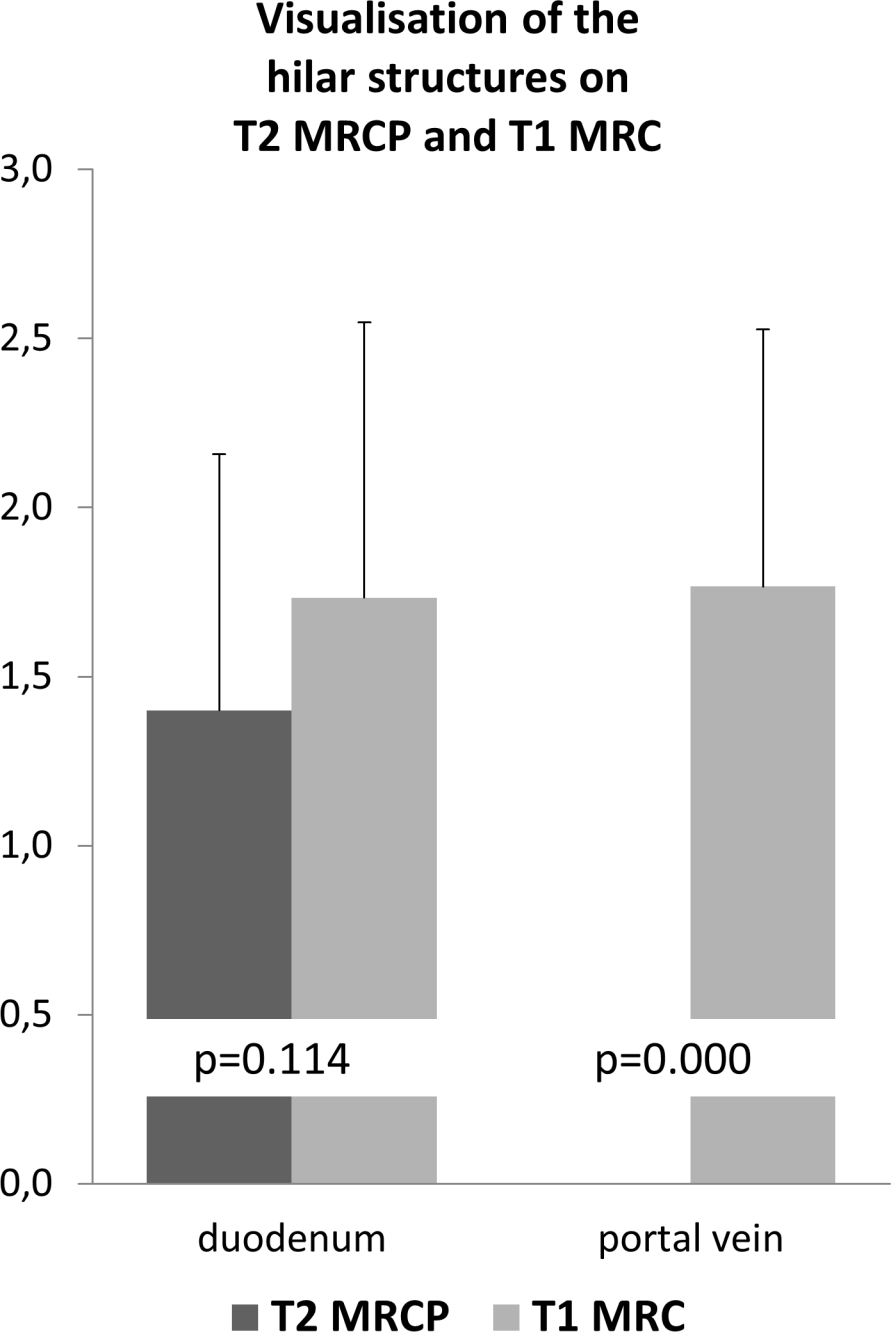
Visualisation of the hilar structures on T2 MRCP and T1 3D MRC. Depiction of the duodenum and thereby relationship of the biliary structures is better with T1 3D MRC. Additionally, the portal vein is confidentially depicted which is not visualised at all on T2 MRCP.

Signal-to-noise-ratio was lower, yet comparable on navigator-triggered T1 3D MRC compared to T2 MRCP (see Table 3 and Fig 5).

**Table 3:** Signal-to-noise-ratio of the central bile ducts on T2 MRCP and T1 3D MRC.

	T1 3D MRC	T2 MRCP	p
common bile duct	29.4	33.6	0.464
common hepatic duct	27.8	36.3	0.137
cystic duct	23.6	27.4	0.446

## Discussion

This preliminary study demonstrated that T1 3D MRC of a specific inversion recovery prepulse has potential to complement T2 MRCP in the diagnostic work-up of the biliary system in patients receiving gadoxetate disodium. Especially for the evaluation of liver structures close to the hilus the T1 3D MRCP showed advantages compared to the T2 MRCP. This was a result of the different acquisition technique of the T1 3D MRC, which is not affected by bowel movement, the relative position of the extrahepatic bile ducts compared to the portal vein and the duodenum.

Standard MRC protocols rely on T2-weighted (T2w) sequences [12, 13]. In the past years, numerous other techniques, including contrast-enhanced sequences have been evaluated, nevertheless, until now, 3D T2 weighted sequences have proven to provide the visualisation of the bile ducts of the highest image quality [10, 11, 14]. This is in line with the findings of our study, as the depiction of the extrahepatic and intrahepatic bile ducts by navigator-triggered T1 3D MRC can be performed with a high image quality, however not superior to T2 MRCP. Especially depiction of the intrahepatic branches was inferior to T2 MRCP, which could be a result of the relatively low concentration of gadoxetate disodium excreted into the small biliary tracts.

Nevertheless, the long acquisition time of the T2 MRCP, due to a long echo time, may lead to deterioration of image quality by bowel movement. In this context different studies have investigated the application of an orally applicated contrast medium that suppresses signal from the intestinal lumen, or butylscopolamin to suppress bowel movement. Especially the effect of butylscopolamin is still discussed and no clear guidelines have been established [15, 16]. Both substances lead to additional costs, and side effects as well as a potential of allergic reactions have to be considered.

To achieve a high image quality using navigator-gated T1 3D MRCP following the administration of gadoxetate disodium no additional medication is required. Another aspect in favour of an additional navigator-triggered T1 3D MRC is that full suppression of the background signal on T2 MRCP does not allow for a relative depiction of the extrahepatic biliary system in relation to structures close to the liver hilus hilar structures, which can be important in cases of external obstruction due to vascular pathologies or neoplasm. In these cases, navigator-triggered T1 3D MRC may give valuable additional information as it allows for a depiction of the adjacent hilar structures, which enables the visualisation of relative positions. Even visualisation of the duodenum, which, as a fluid containing organ, was visualised on most of the studies, was inferior to navigator-triggered T1 3D MRC, as in most of the cases bowel movement reduced the interpretation of the biliary tract on the T2 MRCP. On navigator-triggered T1 3D MRC, interference with bowel movement was much less important, as it is a very fast sequence which highly reduces movement artifacts and bowel content was only depicted by the intraluminal excretion of gadoxetate disodium into the intestine which usually does not interfere with image interpretability.

The results of our study are in line with previous studies. So far, other studies also analysed MRCP data sets using 2D T2 MRCP and contrast-enhanced T1 MRCP [10, 11]. They also found that T2 MRCP enables the visualisation of the hepatobiliary system of a higher image quality compared to T1 MRCP. The key difference between these previous studies in our studies is that we used a specific inversion recovery prepulse to optimise the contrast between the liver and the hepatobiliary system.

Coherent with other studies, navigator-triggered T1 3D MRC is not superior to conventional T2 MRCP in biliary visualisation for biliary evaluation. However, a combination of T2 MRCP and navigator-triggered T1 3D MRC provides significantly better visualisation of biliary structures than T2 MRCP alone [17].

### Limitations

The main limitation is that in none of the patients external hilar compression of the bile ducts was present which might have supported the assumption of navigator-triggered T1 3D MRC being a valuable adjunct to T2 MRCP.

## Conclusion

This preliminary study demonstrated that T1 3D MRC of a specific inversion recovery prepulse has potential to complement T2 MRCP, especially for the evaluation of liver structures close to the hilus in the diagnostic work-up of the biliary system in patients receiving gadoxetate disodium.
